# Implementing a Diabetes Education Program to Reduce Health Disparities in South Texas: Application of the RE-AIM Framework for Planning and Evaluation

**DOI:** 10.3390/ijerph17176312

**Published:** 2020-08-30

**Authors:** Marcia G. Ory, Shinduk Lee, Samuel D. Towne, Starr Flores, Olga Gabriel, Matthew Lee Smith

**Affiliations:** 1 Center for Population Health and Aging, Texas A&M University, College Station, TX 77843, USA; shinduklee@tamu.edu (S.L.); samuel.towne@ucf.edu (S.D.T.J.); matthew.smith@tamu.edu (M.L.S.); 2 Department of Environmental and Occupational Health, School of Public Health, Texas A&M University, College Station, TX 77843, USA; 3 Department of Health Management and Informatics, University of Central Florida, Orlando, FL 32816, USA; 4 Disability, Aging, and Technology Cluster, University of Central Florida, Orlando, FL 32816, USA; 5 Southwest Rural Health Research Center, Texas A&M University, College Station, TX 77843, USA; 6 Coastal Bend Health Education Center, School of Public Health, Texas A&M University, Corpus Christi, TX 78403, USA; starrflores@tamu.edu; 7 Texas A&M South Texas Center-McAllen Campus, School of Public Health, Texas A&M University, McAllen, TX 78503, USA; gabriel@tamu.edu

**Keywords:** chronic disease management, diabetes education, intervention, implementation and dissemination research, South Texas, health disparities, Hispanic, RE-AIM framework

## Abstract

Health disparities in diabetes management and control are well-documented. The objective of this study is to describe one diabetes education program delivered in the United States in terms of the RE-AIM (Reach, Effectiveness, Adoption, Implementation, and Maintenance) Planning and Evaluation Framework. Questionnaires, clinical data, and administrative records were analyzed from 8664 adults with diabetes living in South Texas, an area characterized by high health disparities. The Diabetes Education Program delivered was a professionally led 12-month program involving 8 h of in-person workshop education followed by quarterly follow-up sessions. Changes in average blood glucose levels over the past 3 months (e.g., A1c levels) were the primary clinical outcome. Descriptive and multiple generalized linear mixed models were performed. This community-based initiative reached a large and diverse population, and statistically significant reductions in A1c levels (*p* < 0.01) were observed among participants with Type 2 diabetes at 3 months. These reductions in A1c levels were sustained at 6-, 9-, and 12-month follow-up assessments (*p* < 0.01). However, considerable attrition over time at follow-up sessions indicate the need for more robust strategies to keep participants engaged. For this diabetes education program, the RE-AIM model was a useful framework to present study processes and outcomes.

## 1. Introduction

Health disparities are often geographically bound and occur more frequently in impoverished populations characterized by low socio-economic status and a dearth of available healthcare resources [1,2,3,4]. The U.S.–Mexico border is impacted by extremely high disparities in income, education, and healthcare access, and these social determinants of health make this region among the nation’s highest for chronic disease rates [5,6]. More specifically within this region, the Texas–Mexico border is seen as an area facing significant public health issues, such as higher rates of diabetes [7]. For example, some of the South Texas counties have higher rates of diagnosed diabetes (e.g., 18.8% in Willacy County and 17.2% in Lavaca County) than the statewide (10.9%) or national (9.1%) rates [8]. Given higher rates of undiagnosed diabetes in South Texas [9] and Hispanic population [10], as well as lower accessibility to care [11,12], the diabetes burden is likely to have disproportionately impacted the South Texas region. Adjacent areas in South Texas, especially rural areas, are also experiencing health disparities and lack of adequate healthcare resources [13].

In recognition of the disproportionate disease burden seen in 27 counties in South Texas, the Healthy South Texas initiative was legislatively mandated by the State of Texas in 2015 as a way of addressing the highest impact chronic and acute diseases that have negatively affected the health and quality of life of many residents of South Texas [14]. This approximately USD 10 million per biennium initiative represented a unique partnership between the Texas A&M Health Science Center and the Texas A&M AgriLife Extension System [15].

Rates of diabetes are especially high in South Texas, with an estimated 30% in some counties in the Lower Rio Grande Valley [16]. Given disproportionate diabetes burden in South Texas, a primary point of action for the Health Science Center in the Healthy South Texas initiative was to target diabetes prevention and management in this area. Priority attention was given to this specific disease because of its high prevalence and similar risk factors as other chronic illnesses [17]. Additionally, while uncontrolled diabetes leads to healthcare complications and higher healthcare costs [18,19], diabetes can be managed through lifestyle modifications and healthcare interventions [20,21,22,23,24,25]. Guidance suggests that monitoring and maintaining lower levels of blood glucose helps lower health risk among persons with diabetes [18,26]. Thus, A1c is an important measure when assessing and tracking diabetes management over time.

This study focuses on the Healthy South Texas Diabetes Education Program, which has its origins in more than 20 years of diabetes programming developed by the Coastal Bend Health Education Center [27,28]. Utilizing elements of the RE-AIM (Reach, Effectiveness, Adoption, Implementation, and Maintenance) Planning and Evaluation framework [29], this study describes the program in terms of each of these key elements. Contextual factors will also be discussed to reflect factors contributing to program implementation and outcomes.

## 2. Materials and Methods

### 2.1. Population Setting and Targets

**Figure 1:**
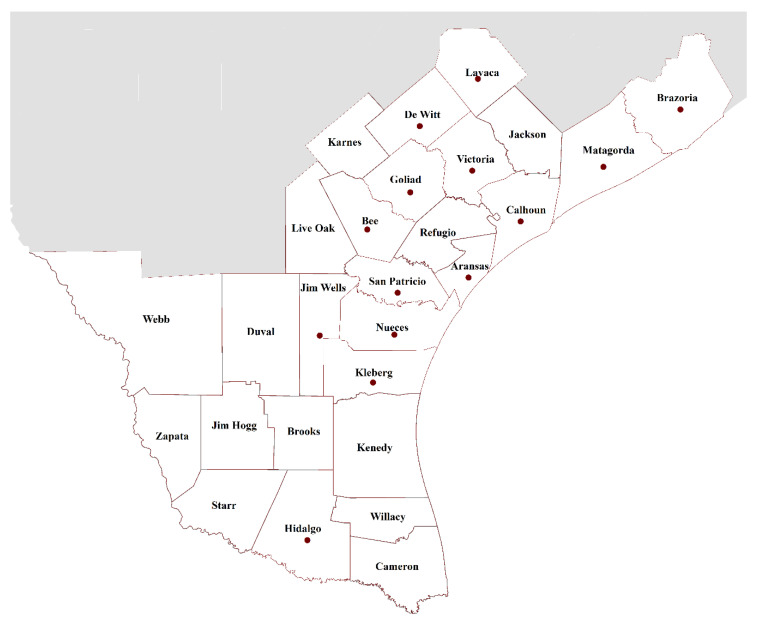
Program coverage area (14 of 27 South Texas counties in which the Diabetes Education Program was offered were marked with a red dot).

Figure 1 illustrated the 27 counties formally included in the Healthy South Texas initiative [30], and the counties in which the Diabetes Education Program was offered were marked with a red dot. Counties along the US–Mexico border were included, as well as areas adjacent to border counties, which were all referred to as South Texas. Including urban, small town, and rural areas, the overall estimated population in these counties in 2015 was approximately 2.8 million, and these areas were among the most impoverished in the nation in terms of socioeconomic status and lack of healthcare services [31,32]. As a community-driven initiative, inclusionary criteria were broad with the intent of serving those both directly and indirectly involved in a person’s diabetes prevention and management. While the focus was on adults with Type 2 diabetes, persons with pre-diabetes and Type 1 diabetes were invited, as well as family members or friends providing care for persons with diabetes. As indicated in Figure 1, the formal Diabetes Education Program was offered in 14 of the 27 South Texas counties in two primary hubs clustered around Nueces and Hidalgo counties.

### 2.2. Recruitment

Given the community-based nature of this initiative and the desire to reach as many participants as possible to show widespread program penetration, participants were recruited from a variety of sources including screenings at health fairs, referrals from healthcare facilities, outreach to community partnerships with flyers and other social media, and self-referrals. Although there was no attempt to standardize referral sources, which differed by organizational sponsorship and location, promotional materials (e.g., flyers) were standardized with a uniform Healthy South Texas brand.

### 2.3. Intervention

The Diabetes Education Program was a recognized American Diabetes Association (ADA) program that was professionally led but also included community health workers for outreach and programming assistance [27]. All ADA-recognized programs provided quality education for people with diabetes and followed the National Standards for Diabetes Self-Management Education and Support (DSMES) guidelines [33]. ADA-recognized diabetes education programs were eligible for reimbursement through many federal and private U.S. insurers [34].

Offered in both Spanish and English, the program consisted of 8 h of face-to-face educational workshop sessions led by at least one trained health professional (e.g., registered nurse (RN), registered dietician (RD), pharmacist, or certified diabetes educator). Workshop sessions were followed with brief (e.g., 15–30 min) in-person individualized follow-up educational and support sessions offered on a quarterly basis for a year. Focal workshop topics included a discussion of, as well as hands-on experiential learning about what diabetes is, blood glucose monitoring, carbohydrate counting, meal planning, reading food labels, medication and insulin administration, preventing diabetic complications, exercise and stress management, and goal setting. The purposes of the follow-up sessions were to check-in with participants regarding their current A1c level and discuss goals and self-management strategies.

### 2.4. Data Collection

These analyses included participants enrolled in the first four years of state funding across two biennia from September 2015 to August 2019 (*n* = 8664). Several self-reported and objective clinical measures were collected at baseline and at each of the four quarterly follow-up appointments by trained program staff. Self-reported data were collected prior to the beginning of the educational workshop via registration forms in English or Spanish, with staff assistance as needed, which provided a portion of the overall baseline data. This study used the following self-reported data: socio-demographic characteristics (age, sex, race/ethnicity, educational level, primary language) and diabetes type.

Controlling blood glucose level was a critical aspect of diabetes management. A1c, which measures average blood glucose over the past 3 months, provided a reliable measure of blood glucose levels that can be used to diagnose diabetes and monitor glycemic control. Thus, A1c values, the primary outcome variable, were collected at baseline and 3, 6, 9, and 12 months after the baseline. A1c was tested using the DCA Vantage Analyzer (Siemens, New York, NY, USA) with valid A1c records ranging from 4% to 14%. The ADA recommended that A1c levels remain below 7% for most adults in general, but appropriate glycemic goal can vary from person to person [26]. ADA also recommended less stringent A1c targets (e.g., <8%) depending on the patients’ health and context [26]. A categorical variable was created based on baseline A1c level reflecting normal (4.0–5.6%), pre-diabetes (5.7–6.4%), and five different levels for those with diabetes, in which higher values represented poorer control and increased health risks (6.5–7.9%, 8.0–8.9%, 9.0–9.9%, 10.0–11.9%, and 12.0% or higher) [18,35].

A secondary biometric measure, body mass index (BMI), was also measured to characterize the study population. Baseline weight was assessed with a professional quality body composition analyzer (TBF-400, TANITA, Arlington Heights, IL, USA). Height was either self-reported or objectively measured by staff using a stadiometer if the person did not know their height. The Tanita automatically calculated the participants’ BMIs based on weight and height measurements. BMI was categorized into underweight (BMI < 18.5), normal weight (BMI = 18.5–24.9), overweight (BMI = 25–29.9), and obese (BMI ≥ 30).

Administrative data included intervention delivery dates and follow-up assessment dates. Attendance estimation for each follow-up only included those who were eligible (i.e., within the time range) to participate in the follow-up. For example, if a participant had their initial appointment in August 2019, then they were not yet eligible for the 6, 9, and 12-month follow-up as of December 2019, and were therefore excluded from the attendance estimation for 6, 9, and 12-month follow-up.

Additionally, program managers provided informal feedback to the Healthy South Texas Office evaluators throughout the study. Programmatic challenges were identified and strategies for improving recruitment and retention discussed.

### 2.5. Analyses

Descriptive statistics (mean and standard deviation or frequency and percentage) were used to describe the characteristics of participants. Not all implementation sites collected the exact same set of variables; thereby, the descriptive statistics reflected available data (i.e., not all variables had the same number of missing cases). Bivariate analyses (e.g., independent t-tests or Chi-square tests) were performed to compare the characteristics of participants recruited during the first biennium and second biennium. Next, retention rates were estimated for each follow-up session. As a part of retention analysis, characteristics of participants who attended and did not attend each follow-up session were described and were then compared using bivariate analyses. This study used data collected between September 2015 and December 2019. Although having four additional data collection months after the second biennium ended in August 2019 allowed for more follow-ups, not all participants had an opportunity to complete all their follow-up sessions. Intervention delivery dates were used to identify and exclude participants from the retention analyses based on their eligibility to participate in the follow-up. For example, participants who participated in the workshop after June 2019 were excluded from the retention analyses for the 6-month follow-up. Similarly, participants who participated in the workshop after March 2019 were excluded from the retention analyses for the 9-month follow-up; and those who participated in the workshop after December 2018 were excluded from the retention analyses for the 12-month follow-up.

Multiple generalized linear mixed models with participant-level random intercepts were fitted to examine changes in A1c level over time among participants with pre-diabetes or Type 2 diabetes. Persons with Type 1 diabetes (*n* = 221) or gestational diabetes (*n* = 12) were not included in the regression models due to small sample sizes. Separate models were performed for the first and second biennia. The first set of models examined changes in A1c levels over time in participants with Type 2 diabetes (*n* = 1922 in the first biennium and *n* = 2733 in the second biennium). The second set of models examined changes in A1c level over time in participants with pre-diabetes (*n* = 380 in the first biennium and *n* = 482 in the second biennium). The third set of models examined any differences in the changes in A1c levels over time based on diabetes type (pre-diabetes or Type 2 diabetes) (*n* = 2302 in the first biennium and *n* = 3215 in the second biennium). The next set of models examined changes in A1c level among participants with Type 2 diabetes by their baseline A1c level (i.e., 4.0–5.6%, 5.7–6.4%, 6.5–7.9%, 8.0–8.9%, 9.0–9.9%, 10.0–11.9%, and 12.0% or higher) (*n* = 1912 in the first biennium and *n* = 2722 in the second biennium). In addition, a separate regression model was used to examine any racial/ethnic differences in changes in A1c level (*n* = 2302 in the first biennium and *n* = 3215 in the second biennium). All regression models controlled for covariates including age, sex, race/ethnicity, education, language, and baseline BMI category. Given that there were only 5 participants reported speaking “Other” as their primary language, they were excluded from the regression analyses. A significance level of 0.01 was used.

### 2.6. Research Ethics

This study involved retrospective reviews and analyses of limited data, and this study was reviewed and approved by the Institutional Review Board at Texas A&M University (IRB2019-0225D).

## 3. Results

Results were presented based on the five RE-AIM elements to provide a case study of this applied research about diabetes self-management education [29].

### 3.1. Reach

Reach was defined as “the absolute number, proportion, and representativeness of individuals who are willing to participate in a given initiative, intervention, or program, and reasons why or why not” [36]. The number of persons who participated in the program and their general characteristics were tracked (Table 1). The majority of program participants were aged between 45 and 64 years (55.3%), female (61.6%), Hispanic (68.6%), and had high school or less education (72.2%). Most participants reported English as their primary language (89.5%) (Table 1). The intervention could be attended by individuals with pre-diabetes or diabetes as well as their family and friends. Among the program participants with a recorded diabetes type, nearly 15% had pre-diabetes and more than 80% had Type 2 diabetes. The mean A1c level was 6.2% among those with pre-diabetes, 8.7% among those with Type 1 diabetes, and 8.6% among those with Type 2 diabetes.

There were statistically significant differences in characteristics of the participants recruited during the first and second biennium (Table 1), indicating changes in program participant profiles and expanded program reach. Compared to participants recruited during the first biennium, those recruited during the second biennium tended to be younger (22.0% vs. 17.1% aged 18–44 years), normal or overweight (32.4% vs. 29.6%), and not knowing their diabetes type (2.0% vs. 0.4%) (Table 1).

#### Retention

In addition to considering initial recruitment, it was important to assess population representativeness over time. The program consisted of an educational session and four quarterly follow-ups to track behavioral goals and clinical outcomes. However, less than 50% of participants attended the first scheduled quarterly follow-up session at 3 months, and the attendance rate for the subsequent follow-up sessions further decreased to 30.5% at 6 months, 23.0% at 9 months, and 18.4% at 12 months. The attendance rates at 9 and 12-month follow-up sessions were higher during the second biennium than during the first biennium (24.8% vs. 21.0% at 9 months and 21.1% vs. 15.9% at 12 months).

Table 2 shows the number and characteristics of overall program participants who attended and did not attend at each follow-up assessment. For all four follow-ups, retention rates were higher among those in the older age group, females, non-Hispanic individuals, those with more than a high school education, and those whose primary language was Spanish (Table 2). Retention rates tended to be lowest for those with BMIs classified as being underweight (Table 2). Among participants with Type 2 diabetes, those not attending a follow-up session at any given time point had significantly higher baseline A1c levels than those who attended the follow-up session (Table 2). Among participants with pre-diabetes or Type 1 diabetes, no statistically significant differences were observed based on baseline A1c level attending a follow-up session at any given time point (Table 2). At the 3-month follow-up, the retention rate was significantly different based on participants’ diabetes type (Table 2). The retention rate was highest among those with pre-diabetes (54.9% at 3 months) and Type 2 diabetes (51.3%), followed by those with Type 1 diabetes (44.3%) and those who were unaware of their diabetes type (35.5%) (Table 2). However, the association between retention rates and diabetes type was not statistically significant at subsequent follow-ups (Table 2).

### 3.2. Effectiveness

Effectiveness was defined as “the impact of an intervention on important individual outcomes, including potential negative effects, and broader impact including quality of life and economic outcomes; and variability across subgroups (generalizability or heterogeneity of effect)” [36]. This study evaluated changes in A1c level among participants from baseline to each follow-up time point. In both the first and second biennium, a statistically significant reduction in A1c level was observed among participants with Type 2 diabetes at the 3-month follow-up (*b* = −0.97, *p* < 0.001 in the first biennium and *b* = −1.13, *p* < 0.001 in the second biennium), and this A1c level reduction was sustained at the 6-month (*b* = −0.98, *p* < 0.001 and *b* = −1.20, *p* < 0.001), 9-month (*b* = −1.10, *p* < 0.001 and *b* = −1.19, *p* < 0.001), and 12-month (*b* = −0.95, *p* < 0.001 and *b* = −1.32, *p* < 0.001) follow-up. For example, for participants with Type 2 diabetes who joined during the second biennium, the average A1c level dropped from 8.6% at baseline to 7.5% at 3 months, and this A1c level reduction was sustained at subsequent follow-ups (7.4% at 6 months, 7.3% at 9 months, and 7.3% at 12 months).

On average, participants with pre-diabetes had A1c levels that remained controlled (<6.5%) from baseline to the subsequent follow-ups. For example, in the first biennium, a statistically non-significant reduction in A1c level was observed among participants with pre-diabetes at all follow-ups (*b* = −0.12 and *p* = 0.06 at 3 months; *b* = −0.13 and *p* = 0.07 at 6 months; *b* = −0.12 and *p* = 0.13 at 9 months; and *b* = −0.12 and *p* = 0.19 at 12 months).

**Figure 2:**
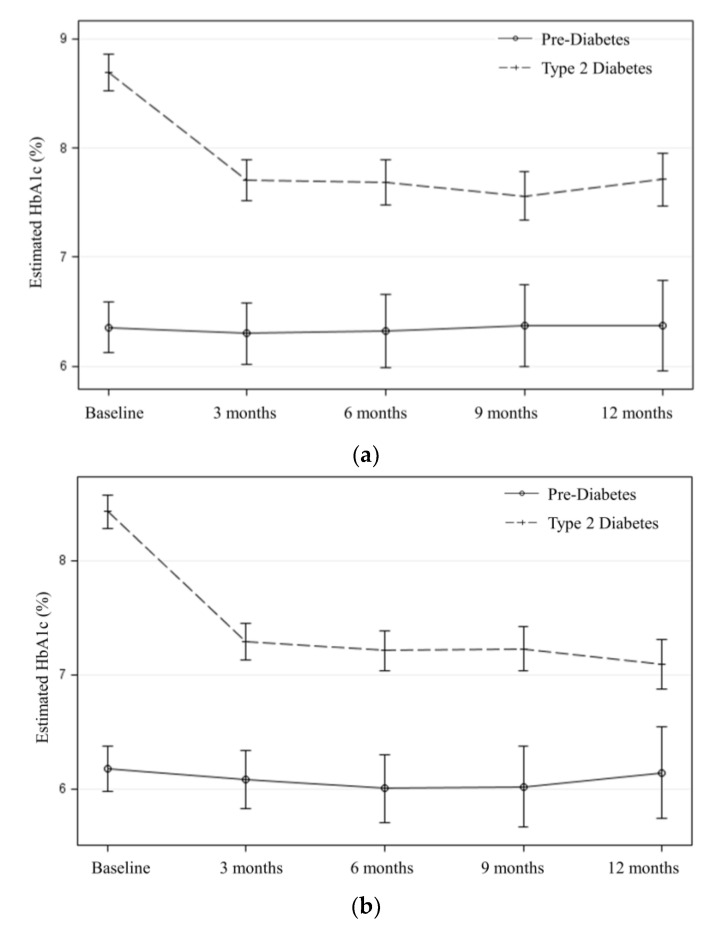
Estimated changes in A1c from baseline to 3, 6, 9, and 12-month follow-up after adjusting for age, gender, ethnicity, education, language, and baseline BMI category, by diabetes type and biennia: (**a**) first biennium; (**b**) second biennium. A1c, average blood glucose level over the past 3 months. BMI, body mass index.

Participants who had Type 2 diabetes showed significantly greater reductions in A1c level than those with pre-diabetes (*p* < 0.001 for the interaction term between time and diabetes type in both the first and second biennia) (Figure 2).

**Figure 3:**
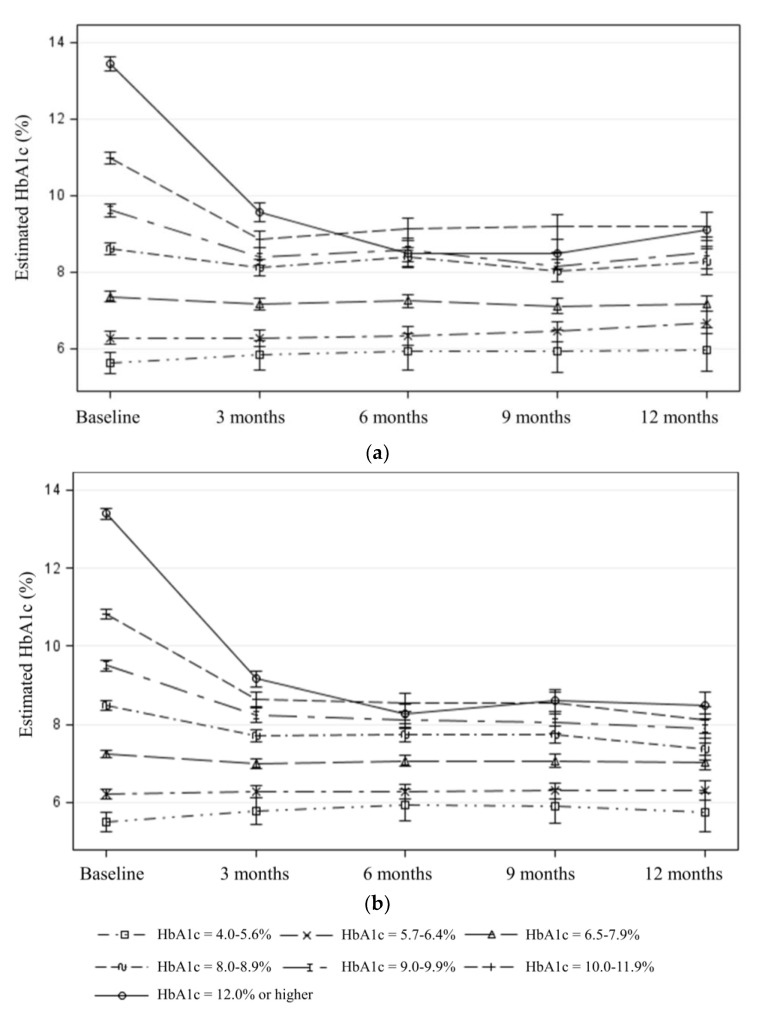
Estimated changes in A1c level from baseline to 3-, 6-, 9-, and 12-month follow-up among those with Type 2 diabetes after adjusting for age, gender, ethnicity, education, language, and baseline BMI category, by baseline A1c and biennia: (**a**) first biennium; (**b**) second biennium. A1c, average blood glucose level over the past 3 months. BMI, body mass index.

Changes in A1c values over time among participants with Type 2 diabetes were also examined based on baseline A1c values. Estimated changes in A1c by baseline A1c values show similar trends over time among the participants enrolled during the first biennium (Figure 3a) and second biennium (Figure 3b). For both biennia, there was a statistically significant modification effect of the baseline A1c level on changes in A1c values over time (*p* < 0.001 for the interaction term between time and the baseline A1c level in both the first and second biennia). Participants with high baseline A1c values (e.g., 8% or higher) achieved a decline in their A1c values at the 3-month follow-up assessments and maintained during the subsequent follow-ups. The estimated A1c level decline was most pronounced for those with highest baseline A1c values (e.g., 12% or higher). On average, participants with controlled diabetes at the baseline remained in control during the subsequent follow-ups.

In a separate regression model, which included the interaction term between time and race/ethnicity, there was no statistically significant differences in changes in A1c values over time among participants with pre-diabetes or Type 2 diabetes (*p* = 0.11 in the first biennium and *p* = 0.25 in the second biennium).

### 3.3. Adoption

Adoption was defined as “the absolute number, proportion, and representativeness of settings and intervention agents (people who deliver the program) who are willing to initiate a program, and why” [36]. In lieu of being able to quantify adoption, the general adoption approach was described. The Coastal Bend Health Education Center (CBHEC) in Corpus Christi served as the Healthy South Texas regional headquarters. The Texas A&M South Texas Center, McAllen Campus, served as a second regional hub. While planning the intervention roll-out, a hub-and-spoke model was determined to be the most effective strategy in which a central “hub” supports multiple “spokes” in communities to provide a range of services. With this approach, CBHEC and the McAllen campus identified other regional partners to help recruit and deliver the program. Due to the staffing requirements (e.g., needing a health professional to lead the educational workshops and sessions), the program was not delivered in all counties; rather, it was more selectively offered around the two hubs—with the spokes representing adjacent service areas.

In accordance with ongoing collaborative health promotion activities in their respective local communities, both CBHEC and the McAllen campus were able to call upon their extended healthcare and public health networks for program delivery assistance. Regional partners that helped deliver the program represented diverse community and clinical entities including community-based organizations, federally qualified health centers, hospitals, clinics, pharmacies, school districts, academic institutions of higher education, state agencies and not-for-profit social service organizations, behavioral health organizations, and city and county government offices. Partnerships varied with some organizations assuming a fuller responsibility for delivering courses on their own, some helping with overall recruitment, and some solely offering physical space for classes.

### 3.4. Implementation

Implementation was defined as “the intervention agents’ fidelity to the various elements of an intervention’s key functions or components, including consistency of delivery as intended and the time and cost of the intervention. Adaptations are also included in this RE-AIM element” [36]. As an ADA-recognized program, there was a need to demonstrate that ADA principles of diabetes education were being followed. This involved an annual review by a designated quality control coordinator to review delivery processes, certify them as compliant, or note aspects to be corrected. Program standardization across sites was facilitated by having a centralized hub for training and data reporting, in coordination with the scientific-administrative oversight functions provided by Healthy South Texas Leadership. CBHEC trained staff in program delivery using a standardized program manual and holding periodic problem-solving feedback sessions with regional program managers and implementers. Adaptations to the program were discussed with the evaluation team to enhance program reach and retention. For example, program staff reported that many participants found it difficult to attend a single-day, 8-h workshop. Therefore, other options were offered such as spreading the workshop over multiple days in two 4-h sessions or four 2-h sessions. Program costs were not tracked in the first biennium, but efforts were made to retrospectively estimate actual program costs based on personnel, supply and space costs toward the end of the second biennium. The program was estimated to cost between USD 800 and 1200 per participant, albeit with substantial variation based on how established the program was delivered at different sites, methods of recruitment, and the number of participants in each class.

A major indicator of program implementation was how many participants engaged in all program activities (e.g., the initial educational workshop plus four quarterly follow-up sessions to track behavioral goals and clinical outcomes over time). As seen in Table 2, only about 50% of participants attended the first quarterly follow-up session at 3 months, and attendance rates decreased for the subsequent follow-up sessions. Given that process evaluation activities were initially built into the program evaluation, program staff were made aware of this issue concerning attendance. Subsequent action was taken in an attempt to bolster follow-up rates. Retention rates at 9 and 12-month follow-up sessions during the second biennium were significantly higher than the rates during the first biennium (i.e., 24.8% vs. 21.0% at 9 months and 21.1% vs. 15.9% at 12 months).

### 3.5. Maintenance

Maintenance was defined at the individual level as “the long-term effects of a program on outcomes after a program is completed” and at the setting level as “the extent to which a program or policy becomes institutionalized” [36]. At the individual level, the trajectory of A1c level change over time during the 12-month intervention period has already been reported in the effectiveness section (Figure 2 and Figure 3). At the setting level, programs included within the Healthy South Texas initiative were intentionally designed to be housed within and delivered by established community partners who could draw upon their existing networks to facilitate programmatic spread and sustainability. For legislative feedback purposes, the amount of actual and in-kind dollars the Health Science Center leveraged during the first two biennia was calculated. From private and public sources, over USD 15,000,000 was identified in direct support and in-kind dollars for the Healthy South Texas initiative (including delivery of the Diabetes Education Program, as well as other disease prevention and health promotion activities) by governmental and nongovernmental entities. The program is still ongoing with a legislative commitment for a third biennium (September 2019–August 2021), and program delivery remains a core function of the two regional hubs.

## 4. Discussion

The Healthy South Texas Diabetes Education Program reached and benefitted large numbers of participants in a region with documented health inequities that have perpetuated health disparities [13]. Utilizing the RE-AIM framework for both planning and evaluation enabled the study team to describe this diabetes program in terms of its reach, adoption, implementation, effectiveness and maintenance [37], and explore the unique challenges faced when applying and assessing RE-AIM elements in community settings [38,39].

This community-based initiative reached a large and diverse population in this region, thus supporting the external validity of the positive results observed. The Hispanic population accounted for over two-thirds of the total population in the service region [40]; hence, the ability to provide the program in Spanish was a critical element that enabled the program to reach this population. An estimated 65,000 persons were served in the first two biennia across a broader range of diabetes outreach and education activities implemented by the Texas A&M Health Science Center Healthy South Texas initiative, in which the Diabetes Education Program was a single component. This initiative capitalized on its understanding of the local community and organizational context, which has been deemed critical for the implementation and dissemination of other health promotion programs [41]. This enabled the implementation sites to draw upon highly visible stakeholders and their diverse relationships with community and clinical organizations for outreach and delivery. Culture, language, and access to resources are known barriers to access to care in the Hispanic population with Type 2 diabetes [42], and collaboration with diverse local community and clinical entities enabled recruitment of culturally competent staff and facilitators to reach the Hispanic population, who are likely to be an underserved population in the region.

This study has generated several general take-home messages to be considered before implementing future health promotion initiatives. Foremost, program planners should conduct community needs assessments and/or engage stakeholders from the communities they wish to target during initial planning, when assessing feasibility, and when deciding which components to include (e.g., considering potential cultural competency considerations and the need to tailor materials). While research studies typically need to offer incentives for participant recruitment [43], the Healthy South Texas initiative promoted program adoption by providing sites with necessary materials and/or subcontracted for services, which allowed programs to be offered free to participants who may have otherwise lacked resources to pay for such services. This was a critical component that helped alleviate or limit the participants’ financial burden as a major barrier to participation, particularly among the socioeconomically disadvantaged population. When considering the generalizability of this evaluation in future settings and populations, program implementers and other key stakeholders should consider this option, where possible, as it may determine whether or not participants can access similar services in other initiatives.

Balancing fidelity to program implementation with the need for adaptation remains challenging in translational research [44]. Over the past two biennia, many of the recommended RE-AIM strategies for improving the implementation processes were implicitly followed and should serve as explicit guideposts in future studies [29,37]. For example, some sites adapted intervention delivery modes and routinized follow-up reminder contacts. Other sites switched the health professional type needed to lead workshops to accommodate local needs and preferences. For example, one hub determined that in their setting a registered dietician was preferable to a registered nurse, and the ADA offered some latitude when selecting the specific type of health professional used to lead workshops without jeopardizing the ADA-recognized status or program fidelity.

Of particular note, the high attrition rates for follow-up visits raises issues about the feasibility and appropriateness of the current Diabetes Education Program structure. The first biennium was a learning curve for the problem facilitators and implementation sites, and additional activities (e.g., reminder calls) were considered and conducted to enhance follow-up rates in the second biennium. Reviewing other successful chronic disease self-management programs [45], the difficulties of expecting the targeted population to consistently engage in intervention sessions over a 12-month period were recognized. Hence, one major adaptation for future programming is to consider modifications of the current Diabetes Education Program and/or the development of a new iteration of such a program with a shorter active intervention period. Long-term supports are still valuable for participant success and should still be incorporated in some capacity (e.g., by their healthcare provider, virtually, telephonically).

The effectiveness of the Diabetes Education Program demonstrated significant decreases in A1c levels over time which were clinically meaningful [46]. However, a closer look provides guidance for future targeting, which is a major concern in intervention research [47]. While reducing A1c levels is a clinical goal for persons with elevated A1c levels, targeting those with A1c values of 8 and above has greater potential to be most cost effective, given these A1c levels are associated with the most diabetes complications and need for costly medical care [19]. Findings from our study indicate that self-management programs adhering to ADA best practices can achieve large decreases in A1c levels among this population.

Maintenance of individual and system-level outcomes is often the most challenging RE-AIM element to achieve [36,48,49]. It is critical to be aware of how context can influence outcomes at both levels. The Practical Robust Implementation and Sustainability Model (PRISM) framework, which is now an integral part of the RE-AIM framework [50], helps us understand the contributory role of recipients, implementation and sustainability, infrastructure, and the external environment. One successful maintenance strategy at the individual maintenance level was to pair the Diabetes Education Program with opportunities for class-based exercise programming throughout the year. Based on feedback from program managers, participants seemed to enjoy these sessions and kept them engaged in the Diabetes Education Program. Additionally, the Diabetes Education Program was seen as a core component of the two Healthy South Texas regional hubs, and organizational efforts are underway to build capacity and support for continued delivery through a network of concerned partners.

In the current third biennium of state funding (September 2019–August 2021), program staff are fully aware of the importance of context during these unprecedented times. Thus, different options for long-term sustainability are being explored, noting that the current COVID-19 crisis may make future state support less likely due to budgetary considerations [51]. Toward this end, strategies for defining and promoting the value proposition of this and other health education programs are being formulated. For example, fee-for-service options, seeking insurance reimbursement for recognized diabetes management programs, and/or providing the program for a modest charge to community or healthcare organizations as a community benefit are being investigated. To assist in making a value proposition based on the potential return on investments made, economic evaluation studies that can demonstrate objective value in multiple ways (e.g., monetary outcomes, measures of gains in quality of life, reductions in years of potential life lost, reductions in potentially preventable hospitalizations) are recommended. Such studies are important to inform local, state, and national stakeholders about the potential return on investment for these and similar studies and should complement other evaluation activities of similar initiatives to reach a broader audience.

### Study Limitations and Strengths

While there were many strengths in this community-based study, there were some limitations that must be acknowledged. This is a case study of a single diabetes education program in a geographic area. Hence, the findings, while promising, may not be generalizable to all community diabetes prevention and control programs or to other areas with differing population characteristics, settings, or varying levels of baseline risk (e.g., A1c level). Additionally, in contrast to academic-based research studies, service delivery often has priority in community-driven programs relative to data collection and management processes, which can limit the types and quality of data collected. For example, although there was a record of engagement by partnering organizations, there was no record of the extent to which organizations agreed to participate when asked. Furthermore, while a large sample size was used, lack of randomization in community settings could be impacted by selection bias.

The RE-AIM model provided a general framework for reporting the planning and evaluation of this initiative. As such, full measures on all RE-AIM dimensions were not collected. While this is a potential shortcoming, it is also aligned with the recognition that applying the RE-AIM model in “real-world” studies does not depend on the assessment of all five dimensions [39].

Despite the relatively large numbers of community residents served by the Diabetes Education Program, the proportion reached relative to people with diabetes residing in these South Texas counties was still minimal, given the high rates of adults with diabetes in the South Texas region [52]. Further, the large attrition rate for follow-up sessions was considerable, which may highlight recruitment or fidelity issues and introduce self-selection and/or a healthful bias (i.e., participants with Type 2 diabetes with lower A1c levels had better retention rates at all follow-up sessions).

Finally, data were not collected longer than 12 months following the Diabetes Education Program workshop; therefore, an assessment of long-term clinical control and management among participants was not possible. Similarly, as the Healthy South Texas Initiative is still ongoing, long-term program delivery and institutionalization post external funding could not be assessed.

However, lessons can be learned from this initiative that advance knowledge about research translation. In line with guidelines for implementing evidence-based diabetes prevention and control programs [53], two successful strategies were employed to enhance reach with the ultimate goal of reducing health disparities in underserved populations. First, community health workers were integral to participant recruitment because they were seen as trusted members in the community [54]. Another successful strategy was establishing and using diabetes health champions [55] (i.e., persons in the program who had successfully lowered their own A1c levels) to serve as program promoters who could engage populations typically characterized as “hard to reach” and who were unaware of, or previously uninterested in, participating in health promotion programs [56].

## 5. Conclusions

The Diabetes Education Program provided an example of dedicated effort to meet the overall public health goal of diabetes prevention and management for all Americans, especially among those experiencing a multitude of social determinants of health inequities [57]. Offering the Diabetes Education Program through two regional hubs was a major advantage for program reach and adoption as well as launching the initiative quickly. Overall, the Diabetes Education Program, as part of the Healthy South Texas initiative, made a substantial impact on the target area reaching diverse and potentially at-risk populations with measured benefits as evidenced in the current study. For long-term programmatic sustainability, such programs will need to be viewed as essential to routine diabetes care. Further studies with longer follow-up periods should be undertaken to examine long-term program effects on participants, as well as any changes and lessons learned in terms of reach, adoption, implementation, and maintenance of a diabetes prevention and management program, such as the Diabetes Education Program.

## Figures and Tables

**Table 1:** Characteristics of program participants.

Characteristics	Overall (*n* = 8664)	First Biennium (*n* = 3514)	Second Biennium (*n* = 5150)	*p*-Value ^a^
Age				<0.001 **
18–44 years old	1720 (20.0%)	594 (17.1%)	1126 (22.0%)	
45–64 years old	4757 (55.3%)	2002 (57.5%)	2755 (53.8%)	
65 years or older	2120 (24.7%)	883 (25.4%)	1237 (24.2%)	
Sex				0.030
Female	5325 (61.6%)	2204 (63.0%)	3121 (60.6%)	
Male	3323 (38.4%)	1297 (37.0%)	2026 (39.4%)	
Race/Ethnicity				0.148
Non-Hispanic White	2215 (25.9%)	930 (26.7%)	1285 (25.4%)	
Non-Hispanic Black	298 (3.5%)	111 (3.2%)	187 (3.7%)	
Non-Hispanic Other races	175 (2.0%)	80 (2.3%)	95 (1.9%)	
Hispanic	5859 (68.6%)	2357 (67.8%)	3502 (69.1%)	
Education				0.421
High school or less	5553 (72.2%)	2237 (71.7%)	3316 (72.5%)	
More than high school	2141 (27.8%)	884 (28.3%)	1257 (27.5%)	
Primary language				0.277 ^b^
English	7746 (89.5%)	3153 (90.0%)	4593 (89.2%)	
Spanish	904 (10.4%)	351 (10.0%)	553 (10.7%)	
Other	5 (0.1%)	1 (0.03%)	4 (0.1%)	
BMI categories				0.007 *
Underweight	432 (5.0%)	198 (5.6%)	234 (4.5%)	
Normal	777 (9.0%)	285 (8.1%)	492 (9.6%)	
Overweight	1928 (22.3%)	755 (21.5%)	1173 (22.8%)	
Obese	5527 (63.8%)	2276 (64.8%)	3251 (63.1%)	
Diabetes type ^c^				<0.001 **
Pre-diabetes	999 (14.7%)	425 (15.4%)	574 (14.3%)	
Type 1	221 (3.3%)	81 (2.9%)	140 (3.5%)	
Type 2	5463 (80.5%)	2238 (81.1%)	3225 (80.1%)	
Gestational	12 (0.2%)	5 (0.2%)	7 (0.2%)	
Do not know	93 (1.4%)	12 (0.4%)	81 (2.0%)	
Baseline A1c (%) (mean (standard deviation)) ^d^				
Pre-diabetes	6.2 (0.97)	6.1 (0.85)	6.2 (1.05)	0.024
Type 1	8.7 (2.17)	8.4 (2.00)	8.9 (2.25)	0.120
Type 2	8.6 (2.25)	8.5 (2.23)	8.7 (2.27)	0.616

* *p* < 0.01; ** *p* < 0.001; ^a^. *p*-values from bivariate analyses (e.g., independent *t*-tests or Chi-square tests) comparing the characteristics of participants recruited during the first biennium and second biennium; ^b^. Chi-square comparison was performed after excluding “Other” language (*n* = 8650); ^c^. In total, 21.7% of diabetes type records were missing, and frequency and percentage were calculated based on available data (*n* = 6788); ^d^. Mean and standard deviation of baseline A1c percentage measures were estimated among those with pre-diabetes and Type 1 and Type 2 diabetes. BMI, body mass index. A1c, average blood glucose level over the past 3 months.

**Table 2:** Characteristics of the program participants who attended and did not attend 3-, 6-, 9-, and 12-month follow-up assessments.

Characteristics	3 Months (*n* = 8664)			6 Months (*n* = 8234) ^a^			9 Months (*n* = 7534) ^b^			12 Months (*n* = 6798) ^c^		
	Attended (*n* = 4147)	Not Attended (*n* = 4517)	*p*-Value ^d^	Attended (*n* = 2509)	Did Not Attend (*n* = 5725)	*p*-Value ^d^	Attended (*n* = 1736)	Did Not Attend (*n* = 5798)	*p*-Value ^d^	Attended (*n* = 1252)	Did Not Attend (*n* = 5546)	*p*-Value ^d^
Age			<0.001 **			<0.001 **			<0.001 **			<0.001 **
18–44 years old	614 (35.7%)	1106 (64.3%)		296 (18.0%)	1349 (82.0%)		188 (12.5%)	1318 (87.5%)		120 (9.0%)	1212 (91.0%)	
45–64 years old	2334 (49.1%)	2423 (50.9%)		1410 (31.2%)	3113 (68.8%)		962 (23.1%)	3200 (76.9%)		703 (18.6%)	3085 (81.4%)	
65 years or older	1177 (55.5%)	943 (44.5%)		791 (39.6%)	1208 (60.4%)		575 (31.9%)	1225 (68.1%)		423 (25.9%)	1210 (74.1%)	
Sex			0.005 *			0.003 *			0.006 *			0.014 *
Female	2611 (49.0%)	2714 (51.0%)		1598 (31.7%)	3447 (68.3%)		1119 (24.1%)	3526 (75.9%)		816 (19.3%)	3408 (80.7%)	
Male	1527 (46.0%)	1796 (54.0%)		905 (28.5%)	2268 (71.5%)		613 (21.3%)	2260 (78.7%)		433 (16.9%)	2125 (83.1%)	
Race/Ethnicity			<0.001 **			<0.001 **			<0.001 **			<0.001 **
Non-Hispanic White	1244 (56.2%)	971 (43.8%)		818 (38.9%)	1284 (61.1%)		594 (30.5%)	1353 (69.5%)		444 (25.3%)	1309 (74.7%)	
Non-Hispanic Black	145 (48.7%)	153 (51.3%)		86 (30.4%)	197 (69.6%)		58 (22.7%)	197 (77.3%)		42 (18.5%)	185 (81.5%)	
Non-Hispanic Other races	81 (46.3%)	94 (53.7%)		47 (28.7%)	117 (71.3%)		40 (26.0%)	114 (74.0%)		33 (23.2%)	109 (76.8%)	
Hispanic	2629 (44.9%)	3230 (55.1%)		1532 (27.5%)	4043 (72.5%)		1027 (20.2%)	4046 (79.8%)		716 (15.7%)	3859 (84.3%)	
Education			<0.001 **			<0.001 **			<0.001 **			<0.001 **
High school or less	2467 (44.4%)	3086 (55.6%)		1413 (26.6%)	3892 (73.4%)		944 (19.5%)	3889 (80.5%)		684 (15.7%)	3665 (84.3%)	
More than high school	1101 (51.4%)	1040 (48.6%)		710 (35.0%)	1316 (65.0%)		512 (27.6%)	1343 (72.4%)		363 (21.7%)	1313 (78.3%)	
Primary language ^e^			<0.001 **			<0.001 **			<0.001 **			<0.001 **
English	3625 (46.8%)	4121 (53.2%)		2166 (29.4%)	5198 (70.6%)		1505 (22.4%)	5220 (77.6%)		1082 (17.8%)	5005 (82.2%)	
Spanish	519 (57.4%)	385 (42.6%)		339 (39.6%)	517 (60.4%)		228 (28.6%)	568 (71.4%)		168 (24.1%)	530 (75.9%)	
BMI categories			<0.001 **			<0.001 **			<0.001 **			<0.001 **
Underweight	157 (36.3%)	275 (63.7%)		86 (21.2%)	320 (78.8%)		52 (13.6%)	330 (86.4%)		25 (7.9%)	293 (92.1%)	
Normal	388 (49.9%)	389 (50.1%)		249 (33.3%)	498 (66.7%)		186 (27.6%)	489 (72.4%)		141 (23.2%)	467 (76.8%)	
Overweight	975 (50.6%)	953 (49.4%)		600 (32.6%)	1241 (67.4%)		431 (25.7%)	1244 (74.3%)		310 (20.7%)	1191 (79.3%)	
Obese	2627 (47.5%)	2900 (52.5%)		1574 (30.0%)	3666 (70.0%)		1067 (22.2%)	3735 (77.8%)		776 (17.8%)	3595 (82.2%)	
Diabetes type ^f,g^			<0.001 **			0.012			0.212			0.116
Pre-diabetes	548 (54.9%)	451 (45.1%)		347 (37.3%)	584 (62.7%)		245 (28.6%)	611 (71.4%)		190 (24.3%)	591 (75.7%)	
Type 1	98 (44.3%)	123 (55.7%)		62 (29.7%)	147 (70.3%)		46 (24.2%)	144 (75.8%)		33 (19.2%)	139 (80.8%)	
Type 2	2801 (51.3%)	2662 (48.7%)		1750 (33.6%)	3456 (66.4%)		1235 (25.9%)	3528 (74.1%)		893 (20.7%)	3430 (79.3%)	
Do not know	33 (35.5%)	60 (64.5%)		11 (20.4%)	43 (79.6%)		4 (16.0%)	21 (84.0%)		2 (16.7%)	10 (83.3%)	
Baseline A1c (%) (mean (standard deviation)) ^h^												
Pre-diabetes	6.2 (1.00)	6.1 (0.94)	0.231	6.1 (0.74)	6.2 (1.05)	0.374	6.1 (0.73)	6.2 (1.01)	0.299	6.1 (0.82)	6.1 (0.94)	0.775
Type 1	8.5 (1.96)	8.9 (2.32)	0.146	8.4 (2.11)	8.8 (2.17)	0.277	8.5 (2.24)	8.7 (2.13)	0.539	9.00 (2.52)	8.55 (2.05)	0.284
Type 2	8.3 (2.19)	8.8 (2.30)	<0.001 **	8.2 (2.13)	8.7 (2.29)	<0.001 **	8.2 (2.16)	8.7 (2.28)	<0.001 **	8.1 (2.12)	8.71 (2.29)	<0.001 **

* *p* < 0.01; ** *p* < 0.001; ^a^. Excluded participants who participated after June 2019; ^b^. Excluded participants who participated after March 2019; ^c^. Excluded participants who participated after December 2018; ^d^. *p*-values from bivariate analyses (e.g., independent t-tests or Chi-square tests) comparing the characteristics of participants who attended and did not attend the follow-up session; ^e^. Excluded 5 participants who reported “Other” primary language to prevent possibility of identifying the individuals; ^f^. Excluded 12 participants who reported having gestational diabetes to prevent possibility of identifying the individuals; ^g^. High missing response rates and frequency and percentage were calculated based on available data (*n* = 6776 at 3 months, 6400 at 6 months, 5834 at 9 months, and 5288 at 12 months); ^h^. Mean and standard deviation of baseline A1c percentage measures were estimated among those with pre-diabetes, Type 1 diabetes, or Type 2 diabetes. BMI, body mass index. A1c, average blood glucose level over the past 3 months.
